# Correction to: “Rode L, Langhoff‐Roos J, Andersson C, Dinesen J, Hammerum MS, Mohapeloa H, Tabor A. Systematic review of progesterone for the prevention of preterm birth in singleton pregnancies.”

**DOI:** 10.1111/aogs.15045

**Published:** 2024-12-19

Rode L, Langhoff‐Roos J, Andersson C, Dinesen J, Hammerum MS, Mohapeloa H, Tabor A. Systematic review of progesterone for the prevention of preterm birth in singleton pregnancies. Acta Obstet Gynecol Scand. 2009;88(11): 1180–9.

To avoid any misunderstanding regarding nomenclature, the authors of this article would like to specify that the term “progesterone” was used in the title, whereas a more appropriate term would be “progestogens.” Similarly, the term “intramuscular progesterone” used in Table III should have been “intramuscular 17α‐hydroxyprogesterone caproate.” There are instances in the text where the terms “17α‐hydroxyprogesterone” and “17α‐hydroxyprogesterone caproate” have been used interchangeably. The authors apologize for these errors.

